# Assessing Human Activity in Elderly People Using Non-Intrusive Load Monitoring

**DOI:** 10.3390/s17020351

**Published:** 2017-02-11

**Authors:** José M. Alcalá, Jesús Ureña, Álvaro Hernández, David Gualda

**Affiliations:** Electronics Department, University of Alcalá, Escuela Politécnica, Ctra. Madrid-Barcelona, Km. 33,600, 28871 Alcalá de Henares, Spain; jmanuel.alcala@depeca.uah.es (J.M.A.); alvaro.hernandez@uah.es (A.H.); david.gualda@depeca.uah.es (D.G.)

**Keywords:** non-intrusive load monitoring, activity monitoring, ambient intelligence (AmI), activity recognition (AR), ambient assisted living (AAL)

## Abstract

The ageing of the population, and their increasing wish of living independently, are motivating the development of welfare and healthcare models. Existing approaches based on the direct heath-monitoring using body sensor networks (BSN) are precise and accurate. Nonetheless, their intrusiveness causes non-acceptance. New approaches seek the indirect monitoring through monitoring activities of daily living (ADLs), which proves to be a suitable solution. ADL monitoring systems use many heterogeneous sensors, are less intrusive, and are less expensive than BSN, however, the deployment and maintenance of wireless sensor networks (WSN) prevent them from a widespread acceptance. In this work, a novel technique to monitor the human activity, based on non-intrusive load monitoring (NILM), is presented. The proposal uses only smart meter data, which leads to minimum intrusiveness and a potential massive deployment at minimal cost. This could be the key to develop sustainable healthcare models for smart homes, capable of complying with the elderly people’ demands. This study also uses the Dempster-Shafer theory to provide a daily score of normality with regard to the regular behavior. This approach has been evaluated using real datasets and, additionally, a benchmarking against a Gaussian mixture model approach is presented.

## 1. Introduction

Life expectancy is becoming higher and higher every year in most developed countries. Although this is clearly a benefit, there are also potential challenges to be faced. For instance, the old-age dependency ratio, the ratio between elderly people and those of working age is rising and expected to double within the next 35 years (from 11.7% to 25.4% of the population). This ratio will exceed 50% in countries such as Japan, Germany, Italy, Spain, and Poland; and the elderly aged 80 years or over will triple in European Union and United States. In fact, this is a challenge on the sustainability of healthcare models that need to care for this community. Furthermore, several studies reveal that the majority of the elderly, at 90%, try to live independently in their own homes, and a shortage of caregivers is expected. Therefore, these two factors, ageing of the population and need of monitoring within the households, are the key issues that have motivated many studies in ambient assisted living (AAL) domain [1].

AAL is based on ambient intelligence (AmI) and it seeks to care for, and monitor, people by adapting their environment and using new technologies. Within this scope, the monitoring of the elderly can be classified into direct and indirect methods and a review can be found in [2,3]. Regarding direct monitoring methods, wearable sensors or body sensor networks (BSN) can be very accurate by registering biomechanical and physiological data, such as blood pressure and heart rate [4]. Due to this accuracy, these sensors are suitable for high-risk subjects that need to be monitored all of the time. For instance, in [5] the author devises flexible graphene electrodes to fully monitor the electrocardiography (ECG) within this community throughout the day. Nevertheless, these sensors are very intrusive, more expensive and, typically, this high level of accuracy is not required for the majority of the population. Consequently, new approaches to indirectly monitor the elderly have been developed, based on the monitoring of activities of daily living (ADLs) by using environmental sensors.

ADL is a term used in healthcare to refer as to the daily activities and routines carried out by people for self-caring. This concept was first proposed by Katz, who stated that age-related diseases have a direct impact on ADLs, and he created ADL indices to measure the dependence level of a person [6]. Furthermore, in [7] instrumental activities of daily living (IADLs) are proposed as more complex activities than ADLs. The performance of these activities is required to determine a successful and independent life.

Concerning the indirect methods and the involved sensors, a good review of previous works is presented in [1,2,8]. Typically, heterogeneous sensor networks are deployed avoiding CCTV cameras and other invasive systems that could threaten the privacy of householders. Hence, there is a mixture of sensors, such as pressure sensors [9], infrared sensors, sound sensors, magnetic switches, optical and ultrasonic sensors, and inertial sensors from smartphones; all of them aiming to capture the human activity within a household. For instance, in [10] inertial sensors are placed in shoes to measure the gait and to foresee the performed ADLs. The procedure requires an activity recognition (AR) algorithm to group event sequences by activities [11]. Then, these activities are assigned to the major ADL activities and they are evaluated to get the ADL indices, also known as circadian rhythms. The composite of major activities based on minor activities, as well as their tracking, have been studied in [12,13]. In [14] a “tape and forget” method to deploy sensors inside the household is presented. In [15] event sequences from heterogeneous sensors are grouped by using support vector machine (SVM). Furthermore, activities can be overlapped over time, which makes the AR clusterization process more difficult. In [16] probabilistic models, such as naïve Bayesian (NB), hidden Markov model (HMM), and hidden semi-Markov model (HSMM), are evaluated to avoid overlapping; whereas in [17] a layered hidden Markov model is used to model the different durations of activities. Further, sensor fusion techniques, such as the Dempster-Shafer theory, are used for AR to determine whether event sequences are overlapped [18,19]. Smartphones are also very compelling to be applied as sensors and in tracking activities [20]. Moreover, activity recognition of ADLs is not sufficient to develop a healthcare monitoring system, it is also necessary to know how activities change over time, which is also a complex task, known as behavior change detection (BCD). In [21] activities are extracted from a heterogeneous sensor network and featured by duration of the activity, sensor density and amount of movement. Then, using two time windows, changes on those features are obtained by means of a permutation-based change detection in activity routine (PCAR) approach and a virtual classifier (VC) approach. Likewise, in [22] daily activity curves are obtained by averaging duration time of those activities over a time window (e.g., a month). Then, using PCAR, a score of the normal daily routine is given.

Overall, AR based on heterogeneous sensor networks presents three major issues that lead to a low implementation in real scenarios [11]: overlapped activities, heterogeneous duration, and the deployment of a complex WSNs, which, in most cases, is intrusive. To increase the acceptance, sensor minimization, low maintenance, and simplicity is pursued. In [23,24] a new approach of monitoring ADLs through electrical signatures of appliances captured by plug-meters is proposed. This monitoring method has been well accepted by subjects, as well as by professionals, during experiments since it is less intrusive. Additionally, daily activities are strongly connected to the usage pattern of appliances and, consequently, they are a good way to infer the human activity. Electrical events are mapped over daily activities, and ADL indices and circadian rhythms are obtained and presented as a decision support tool for experts. The appliance usage patterns are again proved to be correlated with ADLs [25], where a method known as latent dirichlet allocation (LDA) for text analysis is used to map appliance events with ADLs.

In alignment with this, the widespread installation of smart meters is well underway and this has also boosted the development of a new machine learning paradigm: non-intrusive load monitoring (NILM). This is the process of disaggregating the total electrical consumption from the smart meters into individual appliances [26,27]. The smart meter data are distributed through the home area network (HAN) at a low sampling rate (typically about 0.1 samples per second). Thus, probabilistic temporal models have been developed to predict the appliances states (e.g., on or off), such as factorial hidden Markov models (FHMM) [28] or deep learning [29]. These are called eventless NILM algorithms and achieving high performance is still a challenge. Moreover, at higher frequency rates (i.e., higher than 1 Hz), there are event-based NILM algorithms that achieve better performance. These are compounded by an event detector [30,31,32] and a load identification algorithm [33,34] (i.e., around 90% accuracy).

NILM applied to ADLs monitoring is very compelling since it is a completely non-intrusive system, which is a desired feature for large deployments. Additionally, as was mentioned before, ADLs are strongly related to appliance usage patterns and these can be obtained via NILM. To the best of our knowledge, the first use of disaggregation of smart meter data for health monitoring purposes was carried out in [35]. They performed an iterative time-dependent hidden Markov model to disaggregate appliances based on a priori knowledge of inhabitant’s activities. Then, the disaggregation was carried out and every appliance was assigned to a certain activity that could be monitored. In [36] a log Gaussian Cox process is used to monitor the human activity based on the usage pattern for a single appliance. This approach models daily and weekly patterns, as well as uncertainty. The monitored appliance has to be relevant in the daily routine of the householder and has to be daily and manually operated. In this case, a kettle was chosen in an UK dataset. In previous works, the monitoring of the kettle or the TV set has been considered as a relevant variable to detect changes in routines [37] whereas, in [38], the usage pattern of the kettle and fridge during nighttime is used to detect sleep disorders, characteristic of dementia. A multi-appliance monitoring method is proposed in [39], based on a Bayesian approach, which uses Gaussian mixture models for daily and weekly usage patterns. This proposal merges the pattern analysis of all appliances and provides a single score about the normality of the behavior every day.

This work presents a novel algorithm to monitor the human activity through NILM and to evaluate its performance over time. NILM has been already studied widely and it has different resources, such as datasets [40], released open software [41,42] (i.e., in Python and MATLAB, respectively), and a large set of third-party commercial devices [43]. Hence, the focus here is on the activity monitoring based on already disaggregated data. Instead of directly tracking ADLs by mapping event sequences into main activities, as in previous works [36,39], the indices of usage patterns are used to evaluate the performance and to detect whether the subject has deviated from their routine. Thus, the common problems of tracking activities are avoided: overlapped activities, uncertainty and heterogeneity of activity durations, composite of activities, etc. The aim is to simplify the process and directly use the appliance usage patterns as a proxy for health and welfare, detecting anomalies and changes in the routine patterns, which might be a symptom of potential elderly disorders, and reporting them to relatives and caregivers. It is not intended to achieve the accuracy of BSN or WSN approaches, and it is assumed that false alarms may occur with the proposed method. Nevertheless, the cost of a false alarm is low (i.e., it might result in a phone call to check in the house) compared with the benefit of a completely non-intrusive system that does not need maintenance and can be massively deployed. In contrast to [23,24,36,39], the routine evaluation is completely based on NILM, the usage pattern is extracted from multiple appliances and uncertainty is modeled using the Dempster-Shafer Theory, which is commonly used in fusion sensor data. Furthermore, a thorough analysis using real datasets and the Gaussian Mixture model in [39] as a benchmark is carried out to show the benefits of the proposed approach.

The rest of the manuscript is organized as follows: the model description for the algorithm is presented in Section 2; some results and the comparison between models are shown in Section 3; and, finally, conclusions and future work are discussed in Section 4.

## 2. Model Description

The Dempster-Shafer theory (DST), also known as the evidence theory, is widely used in sensor data fusion. It is a generalization of the Bayesian theory [44], where, instead of probability distribution functions, belief functions or mass functions are handled. The disaggregated consumption data from each appliance can be considered as a reading from an independent sensor, which provides a belief about the normality in the usage pattern for that specific appliance by using a mass function. By means of evidence theory, all beliefs are merged, thus obtaining a general belief about the normality in the use of all appliances. This allows to score daily routines and to detect deviations.

The hypotheses $2^{\Omega }$ in the DST are increased, compared to the hypotheses Ω in the Bayesian theory. For instance, for a presence detector, the hypotheses in the Bayesian domain is the universe $\Omega =\left\{h_{1}\left(Presence\right),h_{2}\left(Non\;presence\right)\right\}$. Unlike, in the DST domain the hypotheses are the power set $2^{\Omega }=\{\emptyset ,\;h_{1}\left(Presence\right),h_{2}\left(Non\;presence\right),\;H_{x}\}$, where ∅ is the empty set and $H_{x}$ is a subset for all the potential combinations, in this case $H_{x}=\left\{h_{1}\left(Presence\right)\cup h_{2}\left(Non\;presence\right)\right\}$. Thus, the parameter *x* models the uncertainty that is hardly represented in the Bayesian Theory by assigning a probability of 0.5.

Applying DST to our case, the power set is $2^{\Omega }=\{\emptyset ,\;h_{1}\left(normal\;pattern\right),\;h_{2}\left(abnormal\;pattern\right),\;h_{3}=\{h_{1}\cup h_{2}\}\left(normal\;or\;abnormal\;pattern\right)$. Then, a belief mass function for every element in the power set has to be defined according to Equation (1). A belief mass function is formally called basic belief assignment (BBA), if it meets Equations (2) and (3). The BBA for a certain hypothesis $h_{1}$ represents the proportion of belief for the current state to be such.

(1) $$m:2^{\Omega }\to \left[0,1\right]$$

(2) m(∅)=0

(3) $$\underset{A\subseteq 2^{\Omega }}{\sum }m\left(A\right)=1$$

Belief (or support) and plausibility define the minimum and maximum of the confident interval for the hypothesis *A*, respectively. Hence, the probability for the current state to be *A* is given by an uncertainty interval described in (4), where belief *bel* and plausibility *pl* are defined in Equations (5) and (6). The belief in *A* is evaluated by adding the belief masses from all the subsets of *A*, whereas the plausibility is the sum of all belief masses of subsets where there is an intersection with the set *A*. For instance, the belief of a pattern to be normal or abnormal is $bel\left(H_{x}=h_{1}\cup h_{2}\right)=m\left(h_{1}\cup h_{2}\right)+m\left(h_{1}\right)+m\left(h_{2}\right)$.

(4) bel(a)≤P(A)≤pl(a)

(5) $$bel\left(A\right)=\underset{B|B\subseteq A}{\sum }m\left(B\right)$$

(6) $$pl\left(A\right)=\underset{B|B\cap A\neq \emptyset }{\sum }m\left(B\right)$$

Let us consider two appliances whose hypotheses are to be merged. For that, the Dempster’s rules of combination are applied as in Equations (7)–(9) for the hypothesis *A*.

(7) $$m_{1,2}\left(\emptyset \right)=0$$

(8) $$m_{1,2}\left(A\right)=\left(m_{1}⊕m_{2}\right)\left(A\right)=\frac{1}{1-K}\underset{B\cap C=A\neq \emptyset }{\sum }m_{1}\left(B\right)m_{2}\left(C\right)$$

(9) $$K=\underset{B\cap C=\emptyset }{\sum }m_{1}\left(B\right)m_{2}\left(B\right)$$

where Equation (7) denotes that the mass function assigned to the empty set must be zero, and Equation (8) defines the new mass function for the hypothesis *A* resulting from merging appliances 1 and 2, where 1−K is a normalization factor. Finally, Equation (9) describes the amount of conflict between the two mass sets.

For instance, considering two appliances X and Y, a certain observation window $T_{i}$, and the power set $2^{\Omega }=\{\emptyset ,\;h_{1}\left(normal\;pattern\right),\;h_{2}\left(abnormal\;pattern\right),\;h_{3}=\{h_{1}\cup h_{2}\}\left(normal\;or\;abnormal\;pattern\right)$, their BBAs are defined in Table 1. The Demptster’s rule of combination, Equations (7)–(9), is applied and the resulting fusion of BBAs is depicted in Table 2. The combination of $h_{1}\cap h_{2}$ and $h_{2}\cap h_{1}$ are conflicts because the current state cannot be normal and abnormal at the same time. Consequently, they are assigned to the empty set. The proportion of belief corresponding to the conflict states is assigned to K as in Equation (9). Lately, the parameter K is distributed to the remaining compatible hypotheses according to Equation (8). Table 3 is obtained by adding equal hypotheses in Table 2, normalizing by K and rearranging. The belief and plausibility are evaluated in Equations (5) and (6) from the mass functions of the fusion. Therefore, Table 3 shows that, for the beliefs of appliances X and Y in Table 1, the probability of a pattern to be normal can be found in the interval $0.89\leq P\left(h_{1}\right)\leq 0.92$, defined by Equation (4) within the window $T_{i}$.

The example above can be applied to more appliances by accumulating their evidence iteratively. Therefore, considering *N* appliances, the masses of evidence from appliances 1 and 2 are fused to obtain a new mass: $m_{T_{i}}$, as shown in Table 3. Then, repeating the process, this new mass is merged with the mass from appliance 3, and so on up to the appliance *N*. This iterative process results in a general belief and plausibility based on the accumulative evidential from the *N* appliances in a certain time interval $T_{i}$. This assures that, over that time, the probability of a pattern to be *normal* is higher than the belief and lower than the plausibility.

Likewise, if we are to evaluate a daily pattern, we should proceed by accumulating evidences over the time intervals. Thus, if a daily time T is divided into *I* periods of time *T_i_* in Equation (10), a general mass function $m_{T_{i}}$ for each interval i can be obtained as explained above. These are accumulated following the same process over time, instead of appliances. This results in the belief and plausibility for the performed daily routine within the household.

(10) $$T=\left[T_{1},\ldots ,T_{i},\ldots ,T_{I}\right]$$

### Basic Belief Assignments and Weighing

**Figure 1:**
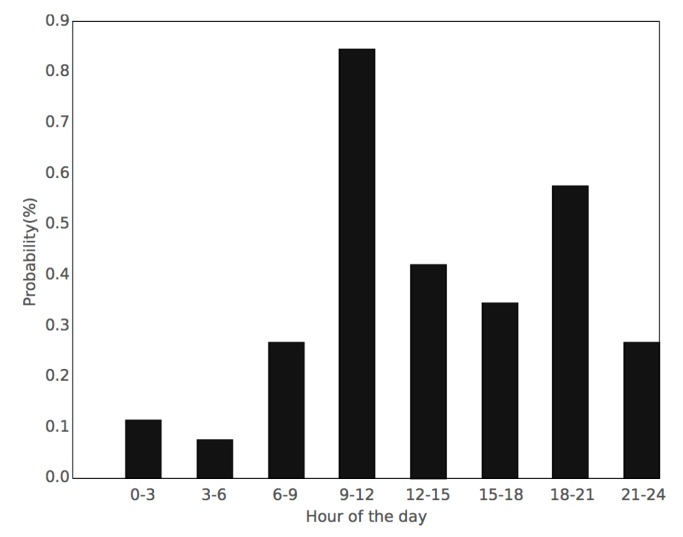
Kettle’s BBA on Mondays with a time interval *T_i_* of 3 h.

Regarding the basic belief functions (BBAs), they are modeled as follows. For every appliance, a BBA is obtained taking into account the day of the week and the time interval $T_{i}$ of the day. It has been empirically proved that the appliance usage patterns not only vary during hours of the day, but it also does in a significant way depending on the day of the week. Likewise, during the training process, the occurrences for each appliance are binned by the time interval $T_{i}$ and the day of the week. The number of occurrences in each bin for a certain day of the week is divided into the total number of that specific day of the week in the training set. This denotes the probability of the appliance to be used at that time of the day for that day of the week. Following this process, similar BBAs to those in Figure 1 are obtained, where the time interval $T_{i}$ was fixed to three hours. Note, for instance, that here the probability $P\left(T_{i}\right)$, where $T_{i}\in \left[9,12\right)$ is the time interval of the day when using the kettle between 9 h and 12 h, is roughly 0.85. Additionally, there is another time interval with high probability of using the kettle between 18 h and 21 h (0.6 approximately). During the test, BBAs are based on this probability, so they are weighed depending on the presence or absence of events within the considered interval $T_{i}$ as in Equations (11)–(13). Thus, the constant $C_{0}$ represents a certainty on the probability $P\left(T_{i}\right)$ for an arrival event, whereas the constant $C_{1}$ shows the certainty in absence of event. The value of these two constants ranges from 0 to 1, and they depend on the application. More details about the certainty constants is discussed in Section 3. In order to meet the requirements in Equations (1)–(3), the mass $m_{T_{i}}\left(h_{3}\right)$ should be defined as in Equation (12) to hold the uncertainty produced by $C_{0}$ and $C_{1}$ in Equations (10) and (11).

(11) $$m_{T_{i}}\left(h_{1}\right)=\{\begin{array}{c} P\left(T_{i}\right)\times C_{0},\;\mathrm{if}\;\mathrm{event}\\ (1-P\left(T_{i}\right)\times C_{1},\;\mathrm{if}\;\mathrm{not}\;\mathrm{event} \end{array}\nabla T_{i}\in \left[0,24\right)$$

(12) $$m_{T_{i}}\left(h_{2}\right)=\{\begin{array}{l} 1-P\left(T_{i}\right)\times C_{0},\;\mathrm{if}\;\mathrm{event}\\ \;P\left(T_{i}\right)\times C_{1},\;\mathrm{if}\;\mathrm{not}\;\mathrm{event} \end{array}\nabla T_{i}\in \left[0,24\right)$$

(13) $$m_{T_{i}}\left(h_{3}\right)=1–(m_{T_{i}}\left(h_{1}\right)-m_{T_{i}}\left(h_{2}\right))$$

The generation of mass functions and basic belief assignments functions is subjective and it does not have to follow this method. For the purpose of this study, the usage pattern of appliances is well represented by the probability of using those appliances within an interval time *T_i_* of the day and, therefore, they model the BBAs. Furthermore, an uncertainty in the model is also desired as the usage pattern could be an indicator of somebody’s healthy routine, but its absence does not necessarily imply the opposite. Thus, the constants $C_{0}$ and $C_{1}$ encode that uncertainty.

## 3. Experimental Results

### 3.1. Datasets, Preprocessing, and Selection of the Training Samples

In order to evaluate the performance of the proposed algorithm, two different datasets have been considered: the Household Electricity Survey dataset [45] and the UK Domestic Appliance-Level Electricity (UK-DALE) dataset [46]. Both are real collected data from the aggregated and disaggregated energy consumption from UK households. The former contains a year data from three single pensioner households, which are the targeted community in this study; whereas the latter one is a two-year collection data from a family household (two adults, two children and a dog). The purpose of presenting the performance over the latter dataset (i.e., a family house) is to enhance the outperformance of the proposed algorithm over the first community (the elderly). As mentioned in Section 1, elderly disorders may imply deterioration in performing daily tasks. Therefore, the performance of elderlies is contrasted against a family house that has a known, strict, and regular routine.

**Figure 2:**
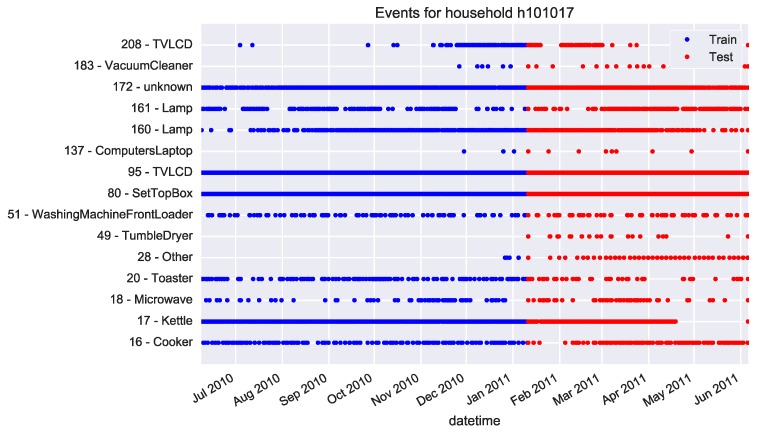
Training and test event samples for household No. 101017 in HES dataset.

The approach presented here is based on the employment of a NILM algorithm and, therefore, only the disaggregated data from datasets are used. Presenting a new NILM algorithm for disaggregation is not the topic of this study and it has been fully studied in previous works already mentioned in Section 1. In this way, the relevant events are extracted from the appliance-level consumption. Relevant events are those whose evaluation can result in the inferring of any human activity inside the household. Consequently, only manually activated appliances with a repetitive pattern over time are considered here, discarding appliances such as the fridge with a continuous and automatic consumption. The relevant events are the recorded timestamp of switching-on for these appliances. Although, the time duration of appliance usages is also a good indication about the routines, this implies a more complex pattern to infer the real human activity, as they could be overlapped. Furthermore, the duration does not really imply any human activity as the appliance could have been left on. Nevertheless, switching-on events do require human activity. For instance, Figure 2 shows the switching-on events in household No. 101017 from the HES dataset over time. Each spot is an event and the *y*-axis represents the appliance code, where non-relevant appliances have been already filtered out. The first 60% of total days are used for training, whereas the remaining 40% is for testing. It is worth noting that some deviation from the routine over time is expected. This is why the model should be trained with earlier days than those used for test, in order to learn the starting *normal* routine. As can be observed in Figure 2, if the ratio between training and test samples is less than 60%, then some appliances do not have enough samples for training.

### 3.2. Definition of Parameters and Constants in DST

Regarding the configuration of the DST algorithm, the observation window $T_{i}$ and the certainty constants $C_{0}$ and $C_{1}$ have been empirically fixed. A six-hour interval has been used for the observation window *T_i_*, so every six hours the presence of each manual appliance (switching-on event) is evaluated and the mass scores in Equations (11)–(13) are obtained. Likewise, the certainty constants are set as follows: $C_{0}=0.9$ and $C_{1}=0.1$. This means that there is a 10% uncertainty ($\left(1-C_{0}\right)\times 100$) in case that a switching-on event of a certain appliance is presented; and a 90% of uncertainty otherwise. Since the *normality* of a pattern is evaluated by switching-on events, it cannot be stated that, if an appliance is not used during a certain time interval, there is an *abnormal* pattern. Note that the human behavior is non-deterministic and, therefore, a high uncertainty is assigned in absence of events. Indeed, there is lack of information, which the DST algorithm models as uncertainty, whereas a Bayesian approach can seldom model it by using only probabilities. The inverse logic can be also applied: the occurrence of an event does not necessarily mean that the pattern is *normal*, but there is more information and that leads to assign only a 10% of uncertainty. These values are empirically obtained and could be changed regarding the confidence on the usage pattern for appliances.

### 3.3. The Benchmark’s Model

In [39] a Bayesian approach is presented to model the usage patterns of appliances by means of Gaussian mixture models and the union probability to score the routine. This model has been improved by dividing the training data into the days of the week, instead of dividing it into working days and weekends. As was proved in [36], some usage patterns have also a weekly repetition, so, for instance, Mondays can be different from Wednesdays. Thus, the accuracy of the GMM model has been improved in comparison with previous results in [39]. This model is used as benchmark in this study.

### 3.4. Analysis of DST and GMM Scores

**Figure 3:**
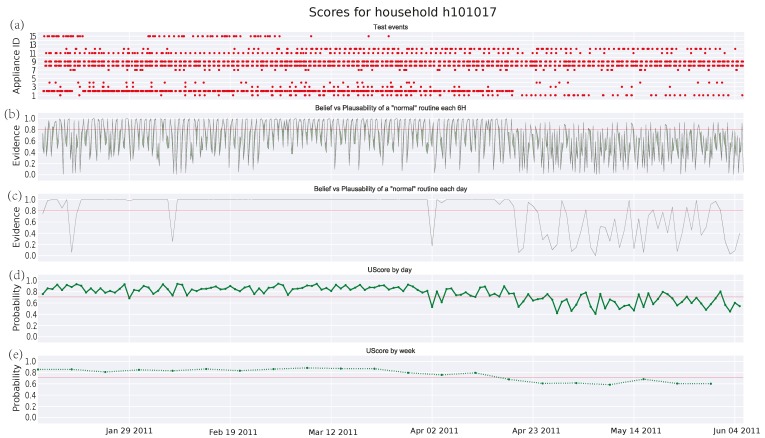
Score for test events in household No. 101017 from HES dataset: (**a**) Test events; (**b**) DST score every 6 h; (**c**) DST score by day; (**d**) union probability score by day; and (**e**) union probability score by week.

**Figure 4:**
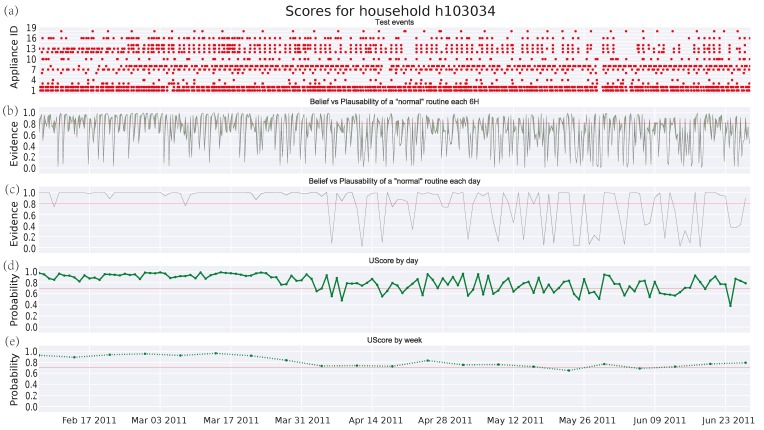
Score for test events in household No. 103034 from HES dataset: (**a**) test events; (**b**) DST score every 6 h; (**c**) DST score by day; (**d**) union probability score by day; and (**e**) union probability score by week.

**Figure 5:**
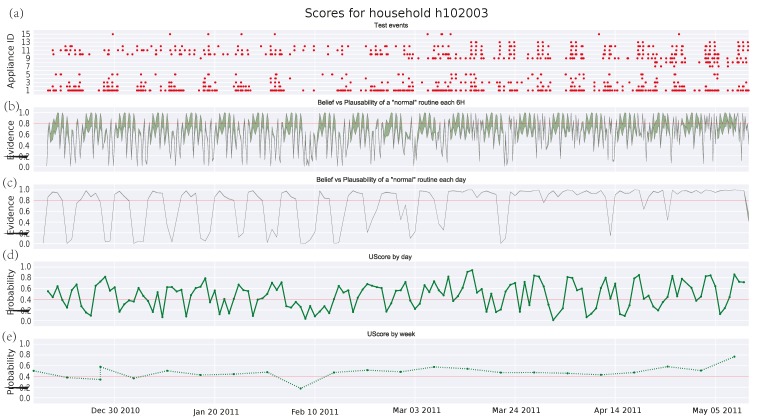
Score for test events in household No. 102003 from HES dataset: (**a**) test events; (**b**) DST score every 6 h; (**c**) DST score by day; (**d**) union probability score by day; (**e**) union probability score by week.

**Figure 6:**
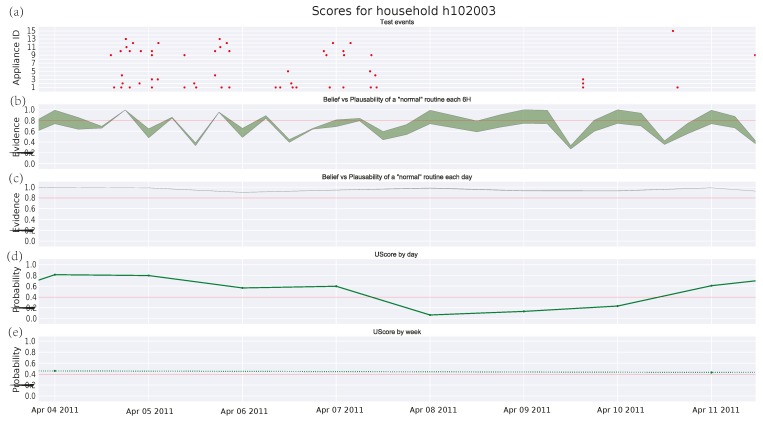
Score for test events in household No. 102003 from HES dataset during a week: (**a**) test events; (**b**) DST score every 6 h; (**c**) DST score by day; (**d**) union probability score by day; and (**e**) union probability score by week.

**Figure 7:**
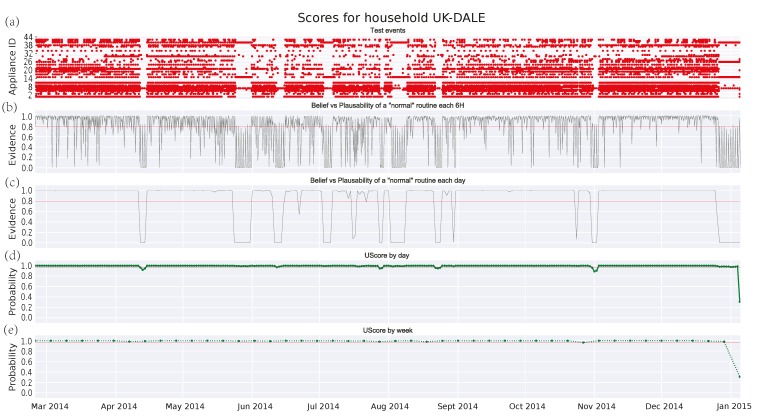
Score for test events in household no. 1 from UKDALE dataset: (**a**) test events; (**b**) DST score every 6 h; (**c**) DST score by day: (**d**) union probability score by day; and (**e**) union probability score by week.

Figure 3, Figure 4 and Figure 5 depict the analysis of the human activity for the three single household pensioners over the test data (145 days roughly in each case) and Figure 6 presents the results for the same house of Figure 5 zoomed to a particular week. Similarly, Figure 7 shows the family household activity during the test (315 days), where it is possible to observe the sensitivity of the proposed algorithm to pattern deviations. Apart from some exceptions, the family house keeps a strict routine and, consequently, the proposed algorithm generates fewer alarms, as shown in the plots in Figures 3a, 4a, 5a, and 6a.

**Figure 8:**
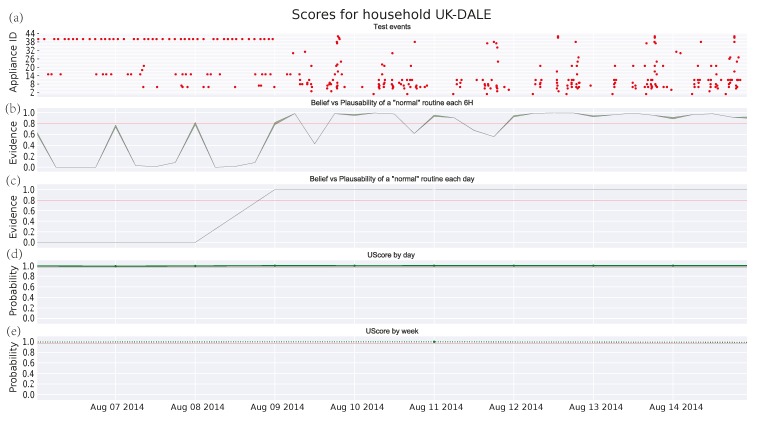
Score for test events in household No. 1 from UKDALE dataset: (**a**) test events; (**b**) DST score every 6 h; (**c**) DST score by day; (**d**) union probability score by day; and (**e**) union probability score by week.

Figure 7a and Figure 8a are similar to the one in Figure 2, where the *x*-axis represents time and the *y*-axis means the different manual appliances, whereas the switching-on events of appliances are drawn as red dots. Due to the fact that, the higher the number of appliances is, the more difficult the labelling of the *y*-axis becomes. Table 4 shows the correspondence between the *y*-axis values and the appliance labels for clarity’s sake. A long-term observation of the score (several months) can help to detect deteriorations in someone’s activity performance. As described in Section 1, these might be a symptom about the apparition of degenerative diseases as dementia, whose early detection could be very beneficial. On the other hand, short-term deviations (several hours or days) are more difficult to interpret, although they should be also watched as they might denote an emergency. Following, some potential cases of deterioration detected by the DST algorithm are presented. Its performance is evaluated in comparison with the GMM algorithm.

#### 3.4.1. Single Pensioner Household No. 101017 in the HES Dataset

Figure 3a shows the test event samples of manually operated appliances that show a repetitive pattern: cooker, kettle, microwave, toaster, lamps, and others. Figure 3b represents the score provided by the DST algorithm with a 6-h observation window *T_i_*, whereas Figure 3c depicts the date score by accumulating evidences over time as was explained in Section 2. In both plots, there is a green filled area limited by the belief and plausibility curves. This is the uncertainty area and it becomes larger as the number of events decreases. The uncertainty is clear in Figure 3b, but the accumulative evidence over the day makes the uncertainty area thinner in Figure 3c, thus obtaining a more accurate score. Figure 3d,e show the results after applying the improved GMM algorithm to score a day, and the average score over the week, respectively. The red line in Figure 3b–e are the empirical thresholds for the DST and the GMM algorithms, where any score bellow those thresholds is considered an anomaly in the behavior. For this specific household, a deviation from the regular routine over months is described with a lower score in both algorithms. These types of long-term deviations are better observed in the DST day score and in the GMM week score. Nevertheless, the former seems to be more sensitive to this kind of pattern, thus rapidly dropping to zero what allows an earlier detection of pattern deviations than the one achieved by the GMM algorithm. The DST algorithm is also able to detect three isolated days of *abnormal* activity. Furthermore, it is worth noting that the oscillation in the daily score for the GMM (see Figure 3c) is high and it makes it difficult to configure the threshold to avoid false alarms. From a manual inspection of data, it has been verified that this deviation is due to those appliances related to cooking, such as the kettle, microwave, and toaster, whose occurrences decrease during that period. Consequently, the ADL of feeding might be deteriorated.

#### 3.4.2. Single Pensioner Household No. 103034 in the HES Dataset

Similar deviations can be found in Figure 4c where the deterioration is due to those appliances related to a range of activities: some days caused by cooking appliances as seen before, others due to the use of the laptop, TV set, or the lighting system. This implies more noise for the GMM approach (see Figure 4d) and the decrease of the mean per weeks becomes very slight (see Figure 4e). Again, the threshold in the daily score for the GMM (Figure 4d) is difficult to be set a priori. Furthermore, looking into month intervals such as the one from 29 January to 19 February of 2011 in Figure 3, short-term anomalies can be detected in the DST algorithm, whereas the GMM algorithm does not show these deviations.

#### 3.4.3. Single Pensioner Household No. 102003 in the HES Dataset

Additionally, there is a case in Figure 5c where a single pensioner has recovered his routine after a deviation. The pattern routine carried by the person involves days of inactivity, which cannot be properly modeled by the Bayesian theory resulting in oscillations from *normality* to an anomalous behavior score provided in Figure 5. This can be closely observed in Figure 6, a zooming zone of a week from Figure 5. Starting on Monday 4 April 2011, this inhabitant spends most of their time out during Fridays, Saturdays, and Sundays. This has been previously learned by the DST algorithm from the training data and it is modeled as an increasing of the uncertainty in Figure 6b, which decreases in Figure 6c due to the accumulated evidence. For instance, on 9 April 2011 in Figure 6b, the uncertainty is such that most time the threshold lies into the area between the belief and the plausibility and, hence, one cannot state whether the pattern is *normal* or *abnormal*. Only from 12 h to 18 h it is feasible to conclude that the period is *normal*, since the threshold lies below both belief and plausibility. The GMM algorithm, which is a classical Bayesian network, cannot handle uncertainties, so it assigns a probability that decreases with the lack of activity. This leads to a higher number of false alarms.

#### 3.4.4. Family House in UKDALE Dataset

Finally, a case of a family with two adults, two children, and a dog is analyzed in Figure 7. Firstly, it is worth noting the increasing number of events coming from different (manually operated) appliances. Furthermore, the scores provided by Figure 7c–e reflect a very strict routine. This routine does not deteriorate over time and only shows *abnormal* intervals that may correspond with holidays or absences in the house. However, these deviations remain undetected by the GMM algorithm. Regarding the threshold, this can be easily set for the DST algorithm, whereas it becomes more difficult for the GMM one. Anyway, it should be close to 1 in order to detect some anomalies.

Zooming into a week period as in Figure 8, it is shown that the DST algorithm is sensitive to the anomalies, whereas the GMM cannot detect them. Since the score in GMM is a union probability, a single appliance which keeps a strict routine can saturate the score and, consequently, masks other anomalies in the remaining appliances. This does not happen in DST where the accumulative evidence of the hypotheses with *abnormal* pattern is higher than the evidence of *normal* patterns. Therefore, it properly models these effects. Furthermore, it is worth noting that the uncertainty is smaller in Figure 6b than its corresponding in Figure 3b, Figure 4b, and Figure 5b due to the increasing number of appliances per 6-h observation window.

Therefore, experimental results show that the DST algorithm behaves better than the GMM algorithm in detecting not only short-term but also long-term deviations. Additionally, the lack of activity is muffled by the uncertainty as in Figure 6, whereas the high activity does not mask other anomalies in the pattern (see Figure 8).

## 4. Conclusions

This paper presents a novel approach for activity monitoring of the elderly to detect deviations from their daily routines. Many health problems and welfare issues are directly related to these deviations, thus, it is a powerful tool for experts, caregivers and relatives. Thanks to the use of NILM, the proposed approach is non-invasive and, therefore, it enables the possibility for larger deployments, which have always been an issue in ADL monitoring systems. The obtained experimental results are promising and they point at the Dempster-Shafer Theory as a very suitable method to score the non-deterministic human behavior thanks to the encoding of uncertainty. Further, modelled uncertainty in the DST algorithm is suitable to be used along with low-performance NILM algorithms (i.e., as those discussed in Section 1) because, in the case of missed events, the uncertainty increases, but the normality score does not decrease. Thereby, its performance has been evaluated with real data and benchmarked with a Bayesian approach, concluding that the DST algorithm outperforms in the detection of short-term as well as long-term routine pattern deviations. The proposed DST approach shows to be more sensitive to pattern deviations and less susceptible to false alarms due to long periods of inactivity. Furthermore, the case of a family house, where a strong routine, is followed is presented to enhance the applicability in scenarios where there are deteriorations and deviations, mostly caused by elderly disorders but not exclusively by them.

Comparing with the previously mentioned BCD algorithms, the approach presented here has the novelty and the advantage of avoiding the use of many sensors and, consequently, the use of AR approaches, whose weaknesses have been pointed out in Section 1, thus the potential scalability is higher. Nevertheless, this also brings drawbacks: there are less information than in other BCD approaches where the duration of the activity and the amount of movement are also analyzed. In conclusion, the proposal is suitable to carry out a coarse monitoring over most elderly persons at large scales, although other BCD methods should be used in order to improve accuracy. In future studies, it is planned to compare the classical BCD methods with the one proposed here; for that, it is necessary to use a dataset similar to [47], merging not only health events but also energy consumption information.

## Figures and Tables

**Table 1:** Basic belief assignments for appliances X and Y (example).

BBAs for X in $T_{i}$	$2^{\Omega }$	BBAs for Y in $T_{i}$
$m_{x}\left(h_{1},T_{i}\right)=0.8$	$h_{1}$	$m_{y}\left(h_{1},T_{i}\right)=0.6$
$m_{x}\left(h_{2},T_{i}\right)=0.1$	$h_{2}$	$m_{y}\left(h_{2},T_{i}\right)=0.2$
$m_{x}\left(h_{3},T_{i}\right)=0.1$	$h_{1}\cup h_{2}$	$m_{y}\left(h_{3},T_{i}\right)=0.2$

**Table 2:** BBA fusion for appliances X and Y.

∩	$X_{h1}$	$X_{h2}$	$X_{h3}$
$Y_{h1}$	$h_{1}\cap h_{1}=h_{1}$	∅	$h_{3}\cap h_{1}=h_{1}$
$Y_{h2}$	∅	$h_{2}\cap h_{2}=h_{2}$	$h_{3}\cap h_{2}=h_{2}$
$Y_{h3}$	$h_{1}\cap h_{3}=h_{1}$	$h_{2}\cup h_{3}$	$h_{3}\cap h_{3}=h_{3}$

**Table 3:** BBA fusion for appliances X and Y.

$2^{\Omega }$	$m_{T_{i}}$	$bel_{T_{i}}$	$pl_{T_{i}}$
$h_{1}$	0.89	0.89	0.92
$h_{2}$	0.08	0.08	0.1
$h_{3}$	0.02	1	1

**Table 4:** Appliance labelling in the *y*-axis by house.

	Appliance Label			
Appliance ID	Household no. 101017	Household no. 103034	Household no. 102003	Family House
1	Cooker	Kettle	Cooker	Boiler
2	Kettle	Microwave	Kettle	Solar thermal station
3	Microwave	Toaster	Microwave	Washer drier
4	Toaster	Cooker extractor fan	Toaster	Dish washer
5		Washing machine	Cooker extractor fan	Television
6	Tumble dryer		Washing machine	Light 1
7	Washing Machine	DVD player	TV-LCD	HTPC
8	Set Top Box	TV-CRT	TV-LCD 2	Kettle
9	TV-LCD	VCR	Computer desktop	Toaster
10	Laptop	Lamp 1	Computer monitor	Fridge freezer
11	Lamp 1	Lamp 2	Printer inkjet	Microwave
12	Lamp 2	Lamp 3	Lamp 1	Computer monitor
13		Lamp 4	Lamp 2	Breadmaker
14	Vacuum cleaner		Lamp 3	Audio amplifier
15	TV-LCD	Vacuum cleaner	Lamp 4	Light 2
16			Lamp 5	Soldering iron
17				Ethernet switch
18			Vaccuum cleaner	Vaccum cleaner
19				Light 3
20				Light 4
21				Light 5
22				Light 6
23				Active subwoofer
24				Light 7
25				Radio
26				Light 8
27				Phone charger
28				Light 9
29				Phone charger 2
30				Light 10
31				Coffee maker
32				Radio 2
33				Phone charger 3
34				Hair dryer
35				Hair straighteners
36				Clothes iron
37				Oven
38				Light 11
39				Baby monitor
40				Light 12
41				Light 13
42				Computer desktop
43				Fan
44				Printer
